# Orbital myiasis: A case report and literature review

**DOI:** 10.1097/MD.0000000000018879

**Published:** 2020-01-24

**Authors:** Yan-Ling Huang, Lu Liu, Hao Liang, Jian He, Jun Chen, Qiao-Wen Liang, Zhi-Yuan Jiang, Jian-Feng He, Min-Li Huang, Yi Du

**Affiliations:** a Department of Ophthalmology; b Department of Hypertension division, the First Affiliated Hospital of Guangxi Medical University; c Guangxi Medical College, Nanning, Guangxi, China.

**Keywords:** Calliphoridae Diptera, Maggot Diptera: myiasis, ophthalmomyiasis, orbital myiasis

## Abstract

**Rationale:**

Myiasis is a parasitic disease caused by fly larvae of the Diptera order that infest human and other vertebrate animal tissues. Orbital myiasis is a potentially destructive infestation of the orbital tissues, which may affect individuals with previous ocular diseases or disorders of consciousness.

**Patient concerns:**

A 72-year-old man presented with a complaint of repeated pain for two years after trauma to his right eyelid and aggravated symptoms with larvae wriggling out for 2 days. An orbital computed tomography scan revealed right eyeball protrusion and periocular soft tissue edema. Two days later, magnetic resonance imaging showed that the shape of the right eyeball was changed and that the normal structure of the eyeball could not be identified.

**Diagnoses:**

Due to the patient's symptoms and imaging examination results, the diagnosis of orbital myiasis was made.

**Interventions:**

The patient was treated by exenteration of the right orbit, and all necrotic tissues and larvae were removed. The defect was repaired via reconstruction with a pedicled musculocutaneous flap from the forehead region. Antibiotics and tetanus toxoid therapy were utilized to prevent potential bacterial infection.

**Outcomes:**

The patient recovered well postoperatively and was discharged uneventfully. During the 6-month follow-up period, the wound healed well.

**Lessons:**

Advanced age and untreated eye trauma are risk factors for orbital myiasis. Timely removal of larvae and elimination of infections are important measures for protecting the eyeball.

## 1 Introduction

Maggots are larvae of Diptera flies, most of which are mainly found in human and animal feces, garbage, decaying plants and animal carcasses and feed on feces and decaying organic matter. In cases of accidents or in certain specific species, they can infest vertebrate animals, including humans, and feed on living or dead tissue, as well as on body fluids,^[1]^ leading to myiasis. Myiasis mainly occurs in animals such as cattle, goats and pigs but occasionally occurs in humans.^[2]^ Advanced age, disease-ridden status, poor self-care, poor hygiene, and rural background are reported risk factors for human myiasis. A pastoral or rural background provides the conditions for the prevalence of myiasis because it is a zoonotic disease. Myiasis is mainly prevalent in tropical and subtropical regions, where a warm and humid climate prevails almost throughout the year,^[1]^ or in developing countries with a large population density and poor sanitation.

Ophthalmomyiasis can involve the eye, orbit, and periorbital tissues. It is classified as external, internal or orbital in accordance with the site of the larvae infestation.^[3]^ Limited superficial infestation of external ocular tissues such as the palpebra and conjunctiva is called external ophthalmomyiasis. When the larvae invade deeply and migrate into the subretinal space, internal ophthalmomyiasis occurs. Orbital myiasis is a more extensive infestation involving orbital tissue and is the most serious form. Once established, orbital myiasis progresses rapidly and can completely destroy the orbital tissues within days.^[4]^ Fortunately, it is the least common form,^[5]^ with only a few cases reported. Management of orbital myiasis ranges from simple manual removal of the maggots to destructive surgeries of the globe and orbit.^[6]^

Medical professionals are unfamiliar with orbital myiasis because it is such a rare disease, which may increase the difficulty of recognition and treatment. Here, we report a case of orbital myiasis and conduct a systematic literature review of cases previously reported in the literature, aiming to better outline the clinical features and therapeutic management of orbital myiasis. The patient himself consented to the publication of the study. This case report was approved by the ethics review committee of First Affiliated Hospital of Guangxi Medical University, (2019-KY-E-036), Nanning, China, and an informed consent form was signed by the patient himself.

## 2 Case report

**Figure 1:**
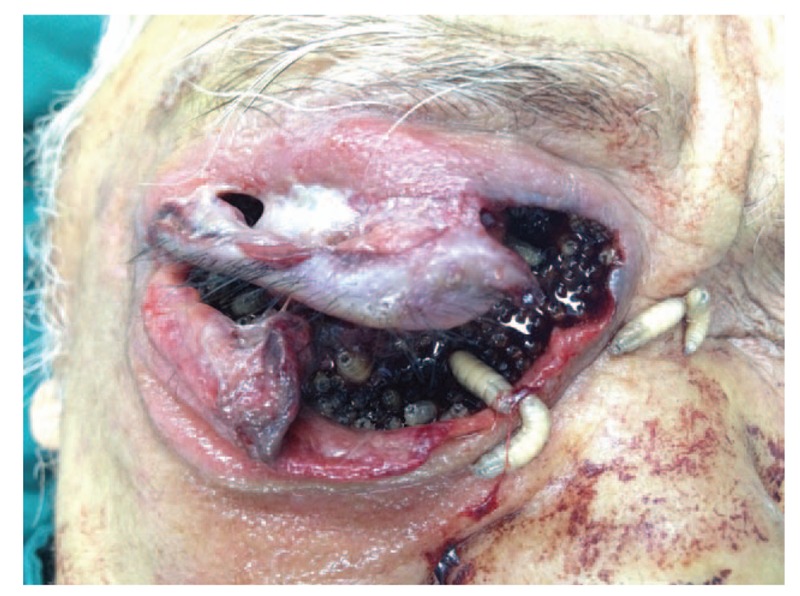
A color photograph demonstrating that the right orbit was destroyed and several larvae were wriggling out.

A 72-year-old male patient presented to the emergency department on October 31, 2015, with a complaint of repeated pain for two years after trauma to his right eyelid and a 2-day history of symptoms aggravated by the wriggling out of larvae. The patient reported that his right upper lid had been injured by cane leaves 2 years prior, but no treatment was received. Then, he experienced repeated pain in his right eye, accompanied by gradually decreased vision until it was completely lost 1 year previously. His painful symptoms worsened 2 days before presentation, with bleeding, a crawling sensation and larvae wriggling out (Fig. 1). He denied a history of alcoholism, previous ocular surgery, or prolonged use of medications.

**Figure 2:**
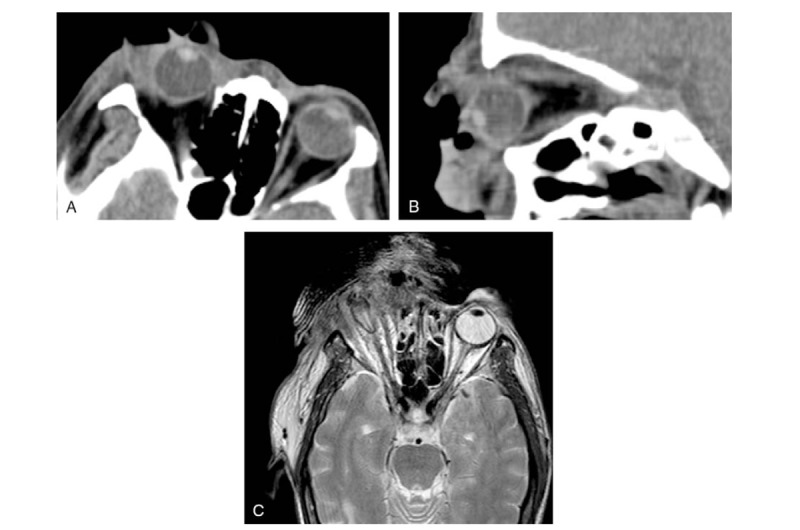
(A and B) An orbital computed tomography scan showed that right eyeball protrusion and periocular soft tissue edema. Two days later, (C) magnetic resonance imaging showed that the shape of the right eyeball was changed and the normal structure of the eyeball could not be identified.

On ophthalmic examination, the visual acuity test revealed no light perception in his right eye. His right periorbital skin was red and edematous, and the eyelid was thickened. There was a large eyelid wound of approximately 4 cm ∗ 1 cm filled with numerous white larvae, some of which were crawling out. No abnormalities were found in the left eye or upon systemic examination. A computed tomography scan revealed that the right eyeball protruded and that the soft tissues around it were swollen (Fig. 2). Two days later, magnetic resonance imaging showed that the shape of the right eyeball was changed and that the normal structure of the eyeball could not be identified (Fig. 2). A diagnosis of orbital myiasis was made.

**Figure 3:**
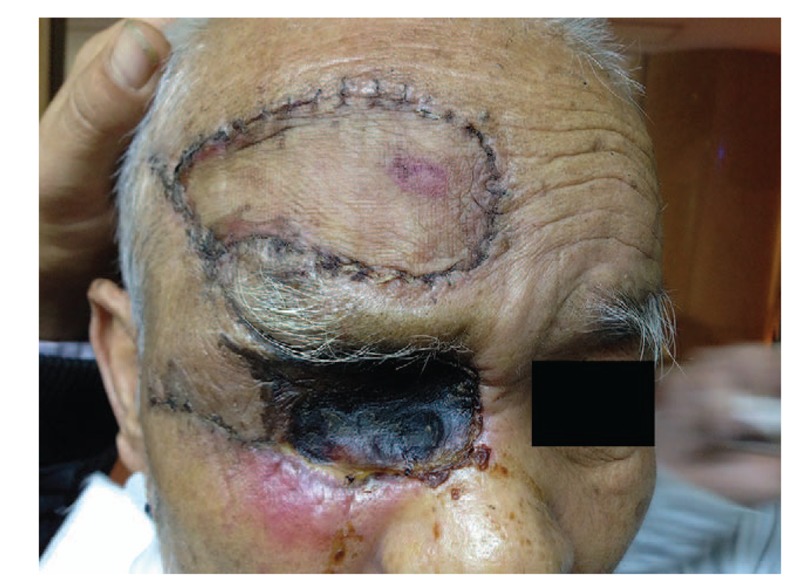
The second week after orbit exenteration and frontal flap reconstruction.

Considering potential infections, the patient received topical levofloxacin eye drops, intravenous ceftazidime and levofloxacin, and a tetanus antitoxin injection. In view of imaging evidence of total destruction of the globe caused by infiltration of the larvae, exenteration of the right orbit was performed in the patient. All necrotic tissues and nearly 100 larvae were removed. Then, the wound was closely observed for infections and possibly missed larvae. Within three days after surgery, there were still 3 larvae crawling out of the orbit. On the ninth postoperative day, the defect was repaired via reconstruction with a pedicled musculocutaneous flap from the forehead region (Fig. 3). The patient recovered well postoperatively and was discharged uneventfully. During the 6-month follow-up period, the wound healed well, and the patient had no further complaints. Subsequently, he was lost to follow-up.

**Figure 4:**
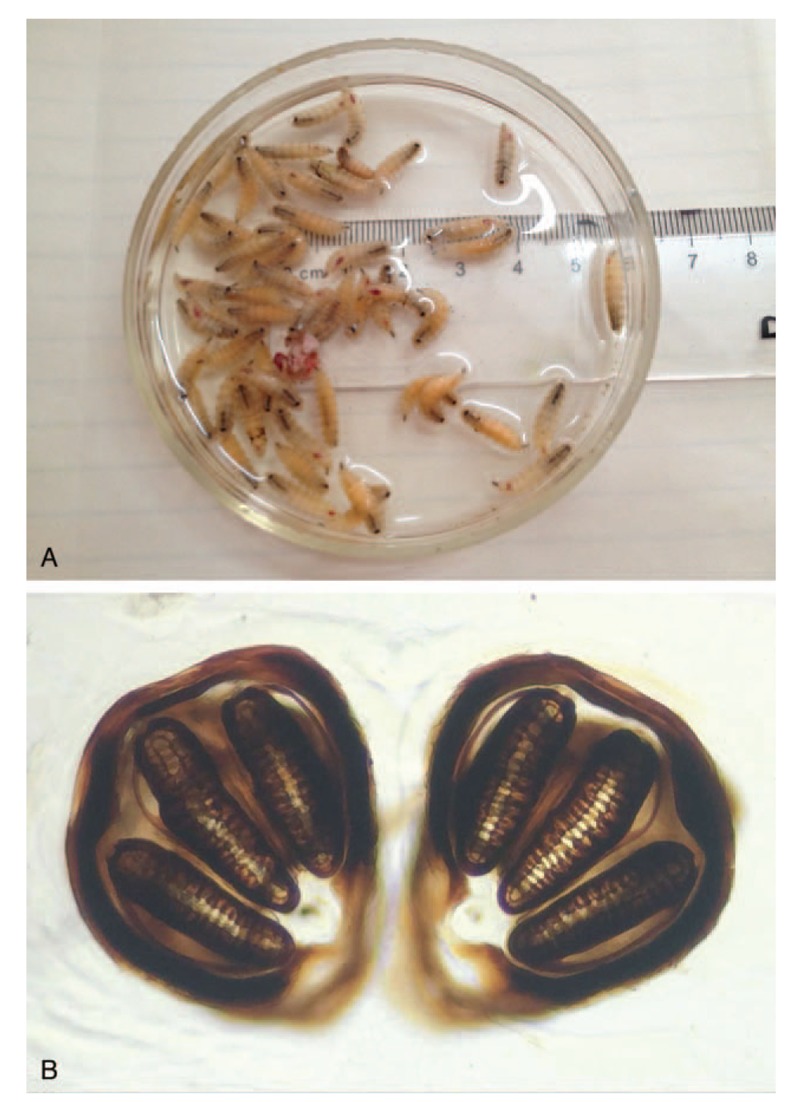
(A) Partial larvae extracted from necrotic tissue after surgery. (B) Posterior spiracle of the larva, 40× magnification.

The histopathological examination of the orbital contents revealed hyperplastic inflammatory granulation tissue, large areas of necrotic tissue and acute inflammatory exudates. The larvae were identified as the larvae of *Lucilia sericata* (Diptera: Calliphoridae) (Fig. 4A and B).

## 3 Literature review

A systematic literature review was performed through a search of the PubMed electronic database to identify all articles regarding human orbital myiasis published between January 1950 and December 2018. References from relevant articles were also included. The investigational strategy was based on an advanced search with the following terms: “orbit” or “orbital” and “myiasis.” Only articles written in English were included.

Forty-six articles were found according to the abovementioned criteria, and their full text was assessed. Twenty-six reports of 27 patients with orbital myiasis were found. After screening the references, no articles needed to be added.

Finally, we identified 27 patients with orbital myiasis reported from 1950 to 2018 in the English-language literature (Tables 1 and 2).

**Table 1:**
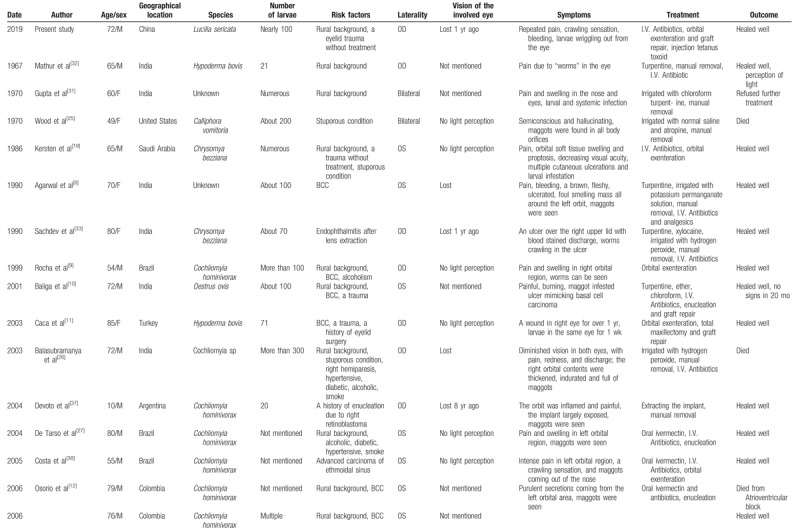
Published reports of orbital myiasis.

**Table 1 (Continued):**
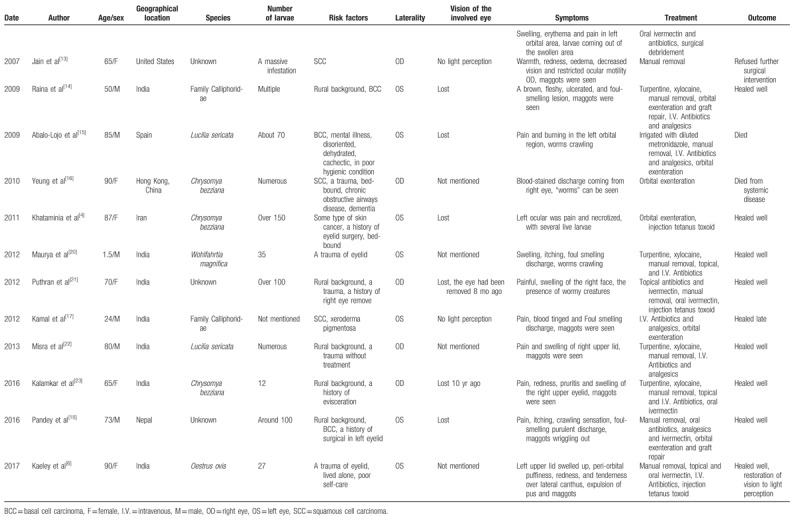
Published reports of orbital myiasis.

**Table 2:**
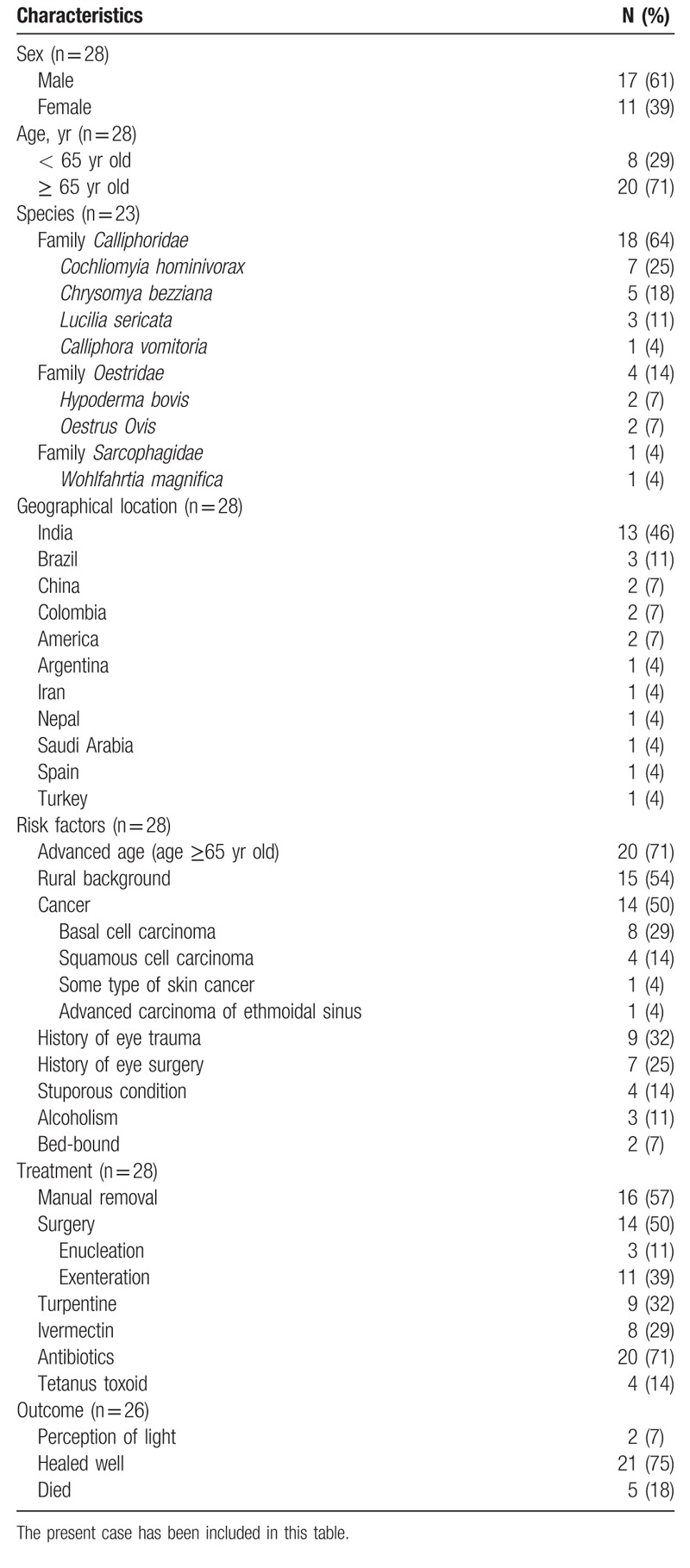
Summary of the 28 cases with orbital myiasis.

## 4 Discussion

According to the literature review, orbital myiasis mainly affects elderly individuals, especially those whose ocular or periocular mucosal barrier is damaged, with causes including trauma, surgery, and ulcers. A rural background is also an important risk factor. The disorder is mainly manifested as inflammatory symptoms, in addition to the symptoms of preexisting diseases. Manual removal is useful for some patients, but half of the cases require surgery because of deep larval penetration and extensive destruction. Early use of ivermectin may avoid surgery, and even if not, such therapy can reduce the degree of damage and the scope of operative intervention.

### 4.1 Causes and risk factors

Diptera is a large order of insects with approximately 150,000 species in 10,000 genera and 150 families.^[7]^ According to the reported literature, there are 6 species of Diptera that cause orbital myiasis. The characteristics of these species are summarized in Table 3.

**Table 3:**
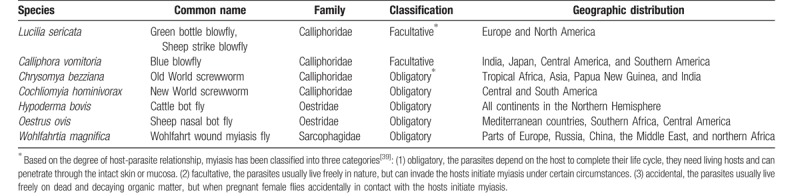
Characteristics of the species reported to cause orbital myiasis.

Cases of orbital myiasis reported between 1950 and 2018 were mainly in Asia and South America, especially India and Brazil. These places are consistent with the distribution of flies mentioned in Table 3. These sites are located in the tropics or subtropics with a warm and humid climate throughout the year and are suitable for the growth and reproduction of flies. Myiasis is a zoonotic disease that is more common in pastoral or rural areas than in urban areas, especially in developing countries lacking basic sanitation and with inadequate garbage disposal. With the increased mobility of people across nations and continents; however, infection may be seen in nonendemic areas.^[5]^

In addition to a rural background, orbital myiasis is mainly associated with malignant tumors, especially skin cancer around the eyes.^[8–18]^ Eye injury without proper medical care is a potential risk factor.^[6,10,11,16,19–22]^ In addition, orbital myiasis is also associated with previous eye surgery, such as evisceration.^[23]^ All of these factors have a commonality of ulcerative or necrotic tissues. Therefore, orbital myiasis is considered to be a special kind of wound myiasis to some extent.^[24]^ Other risk factors include a stuporous condition,^[15,19,25,26]^ alcoholism,^[9,26,27]^ and a bed-bound status.^[4,16]^ Fetid conditions, including excessively long periods of sleep, poor self-care and poor hygiene, may attract flies. Yeung et al^[16]^ reported a case of orbital myiasis occurring in a nursing home. Numerous cases of hospital-acquired myiasis have been reported,^[28–30]^ although these infestations did not take place in the orbital area. The occurrence of these cases indicates that nosocomial infections must be monitored carefully. It is worth mentioning that advanced age is an important risk factor, as orbital myiasis is more common in middle-aged and older patients.

### 4.2 Clinical manifestations

Orbital myiasis is commonly present on only 1 side. It rarely involves bilateral sites. The 2 reported bilateral cases took place in patients who had a systemic infection and were semiconscious.^[25,31]^ The number of infected larvae is usually large (dozens, or even hundreds), and this often indicates a pessimistic prognosis. In addition to the symptoms of tumor ulcers and traumatic wounds from preexisting conditions, the principal clinical manifestations are inflammatory symptoms of redness, swelling, heat, and pain caused by underlying diseases or larvae infection. The itching and crawling sensations caused by the larvae's peristalsis are also common complaints. The examination of most cases shows no light perception, perforation of the globe, and even destruction of the orbit in the diseased eyes. Maggots are seen in the orbital cavity.

### 4.3 Treatments and outcomes

When large numbers of maggots invade deeply into the orbit, they rapidly destroy the eyeball and orbital tissue and then invade the orbital bone, paranasal sinuses, and intracranial tissue. The invasion of the orbital apex most easily penetrates into the brain, causing fatal results.^[26]^ Therefore, early identification and treatment are essential. The treatment principle is complete removal of larvae. Manual removal and surgery are available procedures that should be chosen according to the degree of larval invasion and the extent of tissue destruction, both of which can be used if necessary.

Manual removal should be utilized in the management of less extensive orbital myiasis. Maggots exhibit negative phototaxis. They penetrate deep into tissues to avoid light.^[23]^ Since they can firmly clamp onto tissues by their hook-like structure, forceful removal may result in incomplete extractions leading to an inflammatory response, granuloma formation, and calcification.^[21]^ Therefore, suffocating agents and anesthetic agents are recommended for use before manual removal to make the process easier. Suffocating agents, including turpentine oil,^[8,10,14,20,22,23,31–33]^ petroleum jelly,^[4,23]^ and liquid paraffin,^[4,23]^ can block the larval breathing holes, forcing the aerobic maggots to migrate to the surface for air. It is worth noting that this method may also cause larval death due to asphyxiation in the tissues. Topical administration of anesthetic agents such as xylocaine^[14,20,22,23,33]^ and cocaine^[19]^ can paralyze the larvae and prevent them from penetrating deeper.

Ivermectin, a broad-spectrum antiparasitic drug, is considered a safe and noninvasive means of managing orbital myiasis.^[6]^ The drug promotes the spontaneous emergence of larvae from deep in the orbital tissue and prevents the larvae from causing greater damage, thereby avoiding surgery and reducing the risk of death. Some authors believe that albendazole, butazolidin, and thiabendazole can also play the same role.^[11,23]^

Once the globe is penetrated, enucleation, and even exenteration, is inevitable. In cases where invasion is confined to the globe, enucleation can effectively control the infection.^[10,12,27]^ In cases in which invasion approaches the orbital apex or there is extensive destruction of ocular tissue, exenteration needs to be seriously taken into consideration. In our case, the patient's orbital tissues, including the eyeball, were destroyed by the larvae, and thus, exenteration was inevitable.

Since flies are biologic or mechanical vectors of protozoal, viral, bacterial or helminthic diseases,^[11]^ myiasis may be accompanied by a bacterial infection. Antibiotics and tetanus toxoid therapy are utilized to prevent this complication. However, Puthran et al^[21]^ and Kaeley et al^[6]^ believed that systemic antibiotics were unnecessary because of the antibacterial activity of maggots. Many studies have demonstrated the potent antibacterial activity of larval excretions and secretions of *L sericata* and *Lucilia cuprina* against bacteria.^[34–36]^ However, it is unclear whether other species have this antibacterial activity.

Inflammatory symptoms are relieved after removal of the larvae. Timely treatment can partially restore vision in patients with an intact eyeball.^[6]^ However, the deep invasion of a large number of larvae can destroy the eyeball within a few days, leading to blindness.^[27,33]^ Death occurs in patients who have systemic diseases or are in a stuporous condition at presentation.^[12,15,16,25,26]^ All reported cases had no extraocular involvement.

### 4.4 Preventions

Orbital myiasis can be avoided through proper precautions. Popularizing knowledge is essential. The hazards of flies should be emphasized where flies are prevalent, so that not only myiasis but also other diseases transmitted by flies are prevented. Timely removal of garbage, spoilage plants, and animal carcasses in the environment, as well as a sterile insect technique, can notably reduce fly populations. Adequate nursing home and hospital sanitation and personal hygiene are crucial. Since female flies are strongly attracted by blood and secretions,^[9,11,13]^ accumulated secretions should be cleansed from the eviscerated socket daily; eye trauma requires timely and appropriate medical care, and long-lasting ulcers need medical attention. Moreover, mosquito netting, insect repellents, and insecticides can prevent skin contact.

## 5 Conclusion

Orbital myiasis, a rapidly developing and highly destructive ocular parasitosis, is predominantly caused by the larvae of family Calliphoridae flies, which are prevalent mainly in tropical and subtropical regions. Skin cancer around the eye, untreated eye trauma, and a history of eye surgery are important factors predisposing to orbital myiasis. Advanced age and a rural background are also common risk factors. Timely removal of all larvae can prevent deep larval penetration and protect the eyeball.

## Author contributions

**Conceptualization:** Lu Liu, Hao Liang, Jian He, Jun Chen, Qiao-Wen Liang, Zhi-Yuan Jiang, Yi Du.

**Data curation:** Yan-Ling Huang, Lu Liu, Hao Liang.

**Formal analysis:** Yan-Ling Huang.

**Funding acquisition:** Yi Du.

**Methodology:** Jian He, Jun Chen, Qiao-Wen Liang, Zhi-Yuan Jiang, Yi Du.

**Project administration:** Yi Du.

**Resources:** Lu Liu, Hao Liang.

**Supervision:** Hao Liang, Jian-Feng He, Min-Li Huang, Yi Du.

**Writing – original draft:** Yan-Ling Huang, Lu Liu, Jian He, Jun Chen, Qiao-Wen Liang, Yi Du.

**Writing – review & editing:** Zhi-Yuan Jiang, Jian-Feng He, Min-Li Huang, Yi Du.

Yi Du orcid: 0000-0003-4879-5159.
